# Dystonia type 6 gene product Thap1: identification of a 50 kDa DNA-binding species in neuronal nuclear fractions

**DOI:** 10.1186/s40478-014-0139-1

**Published:** 2014-09-18

**Authors:** Maitane Ortiz-Virumbrales, Marta Ruiz, Eugene Hone, Georgia Dolios, Rong Wang, Andrika Morant, Jessica Kottwitz, Laurie J Ozelius, Sam Gandy, Michelle E Ehrlich

**Affiliations:** Department of Neurology, Icahn School of Medicine at Mount Sinai, One Gustave L Levy Place, New York, NY 10029 USA; Department of Pediatrics, Icahn School of Medicine at Mount Sinai, One Gustave L Levy Place, New York, NY 10029 USA; Department of Genetics and Genomic Sciences, Icahn School of Medicine at Mount Sinai, One Gustave L Levy Place, New York, NY 10029 USA

**Keywords:** Dystonia, Thap1, *DYT6*, Striatum, Cerebellum, Protein

## Abstract

Mutations in THAP1 result in dystonia type 6, with partial penetrance and variable phenotype. The goal of this study was to examine the nature and expression pattern of the protein product(s) of the Thap1 transcription factor (*DYT6* gene) in mouse neurons, and to study the regional and developmental distribution, and subcellular localization of Thap1 protein. The goal was accomplished via overexpression and knock-down of Thap1 in the HEK293T cell line and in mouse striatal primary cultures and western blotting of embryonic *Thap1*-null tissue. The endogenous and transduced Thap1 isoforms were characterized using three different commercially available anti-Thap1 antibodies and validated by immunoprecipitation and DNA oligonucleotide affinity chromatography. We identified multiple, novel Thap1 species of apparent M_r_ 32 kDa, 47 kDa, and 50–52 kDa *in vitro* and *in vivo*, and verified the previously identified species at 29–30 kDa in neurons. The Thap1 species at the 50 kDa size range was exclusively detected in murine brain and testes and were located in the nuclear compartment. Thus, in addition to the predicted 25 kDa apparent M_r_, we identified Thap1 species with greater apparent M_r_ that we speculate may be a result of posttranslational modifications. The neural localization of the 50 kDa species and its nuclear compartmentalization suggests that these may be key Thap1 species controlling neuronal gene transcription. Dysfunction of the neuronal 50 kDa species may therefore be implicated in the pathogenesis of DYT6.

## Introduction

Mutations in *THAP1* (*TH*anatos-*a*ssociated protein domain-containing, *A*poptosis-associated *P*rotein 1) underlie human dystonia type 6 (DYT6) [1-4]. Since the initial reports, many mutations in *THAP1* have been identified in association with both generalized and focal dystonias [5]. Thap1 is a zinc-finger transcription factor that belongs to a family of molecules including over 100 homologues and orthologues [6-10]. Thap1 was first identified in nuclear bodies from primary endothelial cells as a pro-apoptotic Par-4-interacting protein localized in promyelocytic leukemia nuclear bodies [9,10]. The N-terminus of Thap1 contains a zinc-dependent (C2CH) DNA-binding domain that includes a conserved AVPTIF domain, and the C-terminus contains a nuclear localization signal within a coiled-coiled domain [9-11]. *RRM1*, a ubiquitously expressed subunit of ribonucleoside-diphosphate reductase, and *CCAR1* (cell cycle and apoptosis regulator 1) have been identified as direct targets for regulation by Thap1 in endothelial or Jurkat cells. Thus, Thap1 appears to regulate cell cycle proteins and apoptosis [11,12], and of note, dysregulation of transcription and cell cycle proteins is associated with multiple genes, which when mutated, result in dystonia [13].

The Thap1 DNA binding domain (DBD) interacts with an 11-nucleotide consensus sequence 5′-TxxxGGCA-3′ in a target motif known as THABS (Thap1-binding sequence). Most pathogenic missense mutations in *THAP1* occur in the DBD and have either been demonstrated, or are hypothesized, to alter DNA binding [3,14-17]. Other pathogenic missense, nonsense and deletion mutations lead to the production of truncated mRNA species that are either likely subjected to nonsense medicated decay and/or give rise to inactive peptides [3,5]. Importantly, missense mutations have also been identified outside the DBD, and these mutations may alter Thap1 conformation and/or localization in such a way as to indirectly affect the structure and/or function of the DBD. A clear genotype/phenotype relationship has not been established, nor is it definitively known that alterations in transcription of Thap1 downstream targets are responsible for DYT6, as Thap1 may have other, yet to be identified functions.

In order to study the biology of endogenous Thap1 protein, we have applied a series of molecular and immunochemical approaches. The relative molecular mass (M_r_) of authentic exogenous Thap1 was previously established by *in vitro* translation of recombinant c-Myc-tagged human Thap1 protein from a human *THAP1* cDNA. Although the predicted M_r_ is 27 kDa, that recombinant protein had an M_r_ of ~30 kDa as identified by a custom antibody [11]. When specific siRNA sequences were used to silence expression of endogenous *THAP1*, the Thap1-like immunoreactive (T1-LIR) species that were knocked-down in HUVEC cells were also ~ 30 kDa in M_r_ [18]. Gavarini *et al.* (2010) [15] described a T1-LIR species in wild type (WT) mouse brain at ~30 kDa when a commercial rabbit polyclonal anti-Thap1 (Proteintech) was used for immunoblotting. Using the same Proteintech antibody and a second commercial antibody (Novus), Zhao *et al.* [19] identified T1-LIR species in extracts of rat brain tissue and spinal cord at ~ 27 kDa, and their immunoblots also showed a larger, minor T1-LIR species that was not discussed. While Thap1 binding to DNA occurs *in vitro* in monomeric form, the suggestion has been made that *in vivo* DNA binding by Thap1 may require its homodimerization [20]. Sengel *et al.* (2011) [21] used tagged, transfected THAP1 cDNAs to demonstrate homodimerization in HEK293 cells. According to that study, Thap1 homodimerization required the coiled-coil domain. However, an apparent M_r_ for the dimer was not specified. Though these reports focused on species of perhaps similar M_r_, no direct comparisons of co-migration or of knockdown effects were provided, leading to confusion as to whether different laboratories were studying an identical, albeit microheterogenous, species, or whether, instead, various laboratories were studying some mixture of molecules, some authentic and some specious.

Another feature that was studied by the same laboratories was the subcellular distribution of the various T1-LIRs. Nuclear localization of GFP-tagged wild type (WT) Thap1 was observed following transfection of the WT cDNA into human primary endothelial cells [9,10]. Another group employed V5-tagged WT Thap1 and indirect immunofluorescence in a study that revealed signal in both the cytoplasm and the nucleus of U2OS (human osteosarcoma) cells [21]. Alternatively, Lohmann *et al.* [22] reported that transfected GFP-WT Thap1 was exclusively localized to the nucleus in OVCAR-3 (human ovary adenocarcinoma) cells, and that this pattern shifted to include the cytoplasm only when a pathogenic frame-shift mutation was present. Two groups reported that transfected, tagged WT Thap1 protein in HEK293 cells was detected almost exclusively in the nucleus [12,19]. Similar results were observed following transfection of *THAP1* cDNA into T-cell acute lymphoblastic leukemia (T-ALL) human primary cells and in Jurkat cells [12]. As it pertains to dystonia, the key cell type of interest for Thap1 function is of course the neuron, where few observations have been reported. Using the Proteintech antibody, Zhao *et al.* [19] observed that endogenous T1-LIR in rat brain was juxtanuclear in location and was especially evident in the cytoplasm of cerebellar Purkinje cells, and Gavarini *et al.* [15] reported the presence of T1-LIR in nuclear extract from cerebellum, striatum, and olfactory bulb (~30 kDa species).

Herein, we report the application of cDNA transfection, viral transduction, immunoprecipitation and gene silencing strategies in both neuronal and non-neuronal cells so as to yield a more comprehensive analysis of the molecular speciation of endogenous and transfected Thap1. We also employed the advanced techniques of DNA oligonucleotide affinity chromatography and a murine model of *Thap1* deletion *in vivo* (Ruiz, Ozelius, and Ehrlich, unpublished data). In order to standardize our data, we performed side-by-side comparisons of the T1-LIR species detected by 3 commercially available anti-Thap1 antibodies.

## Materials and Methods

### Animals

Mice were individually housed and maintained on a 12:12 light:dark cycle (lights on at 7 am) with *ad libitum* access to food and water throughout the course of the entire experiment. All experimental protocols were conducted within NIH guidelines for animal research and were approved by the Institutional Animal Care and Use Committee (IACUC) at Icahn School of Medicine at Mount Sinai. Whole brains and dissected brain regions were processed immediately or snap frozen and stored at −80°C.

### Cell culture

HEK293T cells were cultured in DMEM (Gibco) supplemented with 10% FCS, Penicillin/Streptomycin and L-Glutamine and placed in a humidified 5% CO_2_ incubator at 37°C. Cells were transfected with pCDNA5.2-V5/Thap1-GFP or pCDNA6.1-Thap1-GFP plasmids using JetPEI (PolyPlus Transfection) at a PEI:DNA ratio of 3:1 following manufacturer’s instructions.

Primary striatal neurons in culture were prepared from embryonic brains as previously described [23]. Cells were transduced one day after plating, either with Ad (adenovirus)-V5/hThap1-GFP, Ad-hThap1-GFP, or Ad-GFP virus preparations (SignaGen Laboratories) at MOI 50–200, or with lentiviral (LV) preparations of pLentilox-mThap1-GFP or pLentilox-GFP control (MOI 5–10). All viral vectors included the CMV promoter. Cells were harvested for protein extraction 72 hours (h) after transduction using either RIPA buffer containing 0.2% SDS and a commercial proteinase inhibitor cocktail (Roche) or cytoplasmic/nuclear extraction buffer or RNA extraction (see below). HEK293T cells were transduced at multiplicity of infection (MOI) 5–20 with adenoviral particles and harvested 24-48 h post-transduction or transduced with lentivirus (LV) (MOI 2–5), and harvested 72 h post-transduction.

Gene knock down experiments were performed in HEK293T cells using ON-TARGET plus siRNA specific to human Thap1 (Dharmacon). Cells were plated in P24-well plates (90,000 cells/well) one day prior to transfection. Immediately before transfection, media were replaced with fresh DMEM with 10% FCS, Penicillin/Streptomycin and L-Glutamine. siRNA was combined with Lipofectamine 2000 (Invitrogen) and added to the wells, achieving a final concentration of 25 or 50 nM. GAPDH siRNA and GL2-Luc irrelevant siRNA (Dharmacon) were used as controls. The siRNA sequences were co-transfected with pCDNA6.1-hThap1 overexpression vector. pGIPZ-shRNA Clone ID: V3LMM_491221 or V2LMM_109696 lentiviral particles were used to knock down murine Thap1 in primary striatal neurons, with non-silencing control shRNA (GIPZ lentiviral non-silencing shRNAmir #RHS4346) at MOI 10 (Thermo Scientific). Knock down experiments in primary striatal cultures were also performed using adeno-associated viral (AAV) particles carrying shRNA specific to murine Thap1 (AAV-shRNAThap1) or irrelevant shRNA (AAV-shRNA scrambled) purchased from Virovek, Inc.

For the Thap1 protein half-life experiments, HEK293T cells were transfected with pCDNA5.2-V5/Thap1-GFP vector and then treated with 50 μg/ml cycloheximide (SIGMA). Cells were washed and extracted in RIPA buffer with proteinase inhibitors at the indicated time points.

### Lentiviral packaging

HEK293T cells were plated 24 h prior to transfection using either 3.5×10^6^ per 100 mm dish or 8×10^6^ per 150 mm dish. On the following day, media were changed to fresh DMEM plus 10% FBS and Penicillin/Streptomycin (Gibco). The corresponding pLentilox or pGIPZ lentiviral backbone was co-transfected along with the auxiliary vectors pCMV-dR8.2 dvpr and pCMV-VSV-G using JetPEI (PolyPlus) at PEI: DNA ratio 2:1. Media were changed at 24 h post-transfection, and supernatants were harvested 48 and 72 h post-transfection. Viral concentration (60-70×) was performed by centrifugation at 4000 g for 30 minutes using Millipore Amicon Ultra-15 30 K tubes.

### Nuclear/cytoplasmic extracts

HEK293T cells or striatal primary cultures were extracted on ice by homogenization of tissues in hypotonic buffer (10 mM HEPES pH 7.9, 10 mM KCl, 2 mM MgCl_2_, 0.1 mM EGTA, 0.2 mM PMSF, protease inhibitor cocktail (Roche), sodium pyrophosphate (0.2 mM), fluoride (10 mM) and orthovanadate (50 mM). Homogenates were incubated for 15 minutes on ice before addition of NP-40 to a final concentration of 0.6% (v/v), and then vortexed. The cytosolic fraction was collected by centrifugation at 5000 × *g* for 15 minutes at 4°C, and the supernatant was removed, aliquoted, and stored at −80°C. The pellet was washed once in hypotonic buffer and resuspended by vortexing in hypertonic buffer (20 mM HEPES pH 7.9, 420 mM NaCl, 1.5 mM MgCl_2_ 0.1 mM EGTA, protease inhibitors as per hypotonic buffer above). The resuspended pellet was incubated for 30 minutes at 4°C with rotation shaking. Nuclear extracts were centrifuged at 13000 *g* for 15 minutes at 4°C, and the supernatants were aliquoted and stored at −80°C.

### Western blotting

Tissues or cells were homogenized in RIPA buffer containing 0.2% SDS, 1 mM EDTA, 1% NP-40, 0.5% sodium deoxycholate, 1 mM sodium orthovanadate, 1 mM sodium fluoride, 1 mM PMSF and EDTA-free mini-complete protease inhibitor cocktail tablet (Roche). The protein concentrations of the extracts were determined using the bicinchoninic acid method (Thermo Scientific). Proteins were separated in 10% or 12% Bis/Trisacrylamide gels (BioRad) and transferred to nitrocellulose membranes. Blocking was achieved with 5% (w/v) milk in PBS. The primary antibodies to Thap1 were rabbit polyclonal (Proteintech) used at 1:2000 dilution, mouse monoclonal 3H3 clone (Santa Cruz) used at 1:1000, and mouse monoclonal NeuroMab clone N325B/65 (UC Davis/NIH NeuroMab Facility) at 1:5 dilution. Additionally, monoclonal mouse anti-V5 (Invitrogen) was used at 1:2000. TorA was detected with ab34540 rabbit polyclonal (Abcam). All primary antibodies were incubated in in PBS-Tween 5% milk for 2 hours at room temperature or overnight at 4°C. Membranes were then washed in TBS-Tween. Secondary antibodies anti-rabbit IgG–HRP (Vector Labs) or anti-mouse IgG-HRP (Vector Labs) were used (1:10.000) in PBS-Tween 5% milk for 1 hour at room temperature. Immunoreactive proteins were visualized using Enhanced Chemiluminiscence (ECL) on a Fujifilm LAS4000 imaging device.

### Real-time quantitative polymerase chain reaction (RT-qPCR)

Primary mouse striatal neurons grown in a monolayer were lysed directly in the cell culture wells and homogenized with a 20-gauge needle. Total RNA purification was performed with the RNeasy Micro kit (Qiagen), and was carried out according to the manufacturer’s instructions. E9 embryos were homogenized in TRIzol® reagent (Invitrogen, Carlsbad, CA) and the RNA was extracted using PureLink™ Micro-to-Midi™ Total RNA Purification System (Invitrogen, Carlsbad, CA) following the manufacturer’s instructions. RNA concentrations were determined with a spectrophotometer. Mouse cDNA was synthesized using SuperScript® III Reverse Transcriptase (RT) (Invitrogen, Carlsbad, CA). Amplification was performed on 20 to 300 ng of cDNA with Taqman gene expression Master Mix (Life Technologies). Semi-quantative real-time PCR was performed on StepOnePlus™ Real-Time PCR System (Applied Biosystems, Foster City, CA) using TaqMan Gene Expression assays: Mm01212603_m1 for mouse *Thap1* and Mm00485870_m1 for mouse *Rrm1* (Ribonucleotide Reductase M1). We normalized expression levels with the assay Mm01151063_m1 for TBP (TATA-binding protein)*,* Mm00607939_s1 for *Actb* (ß-Actin) or Mm00446968_m1 for *Hprt1* endogenous controls (Applied Biosystems, Foster City, CA)*,* which demonstrated low variability in our test samples. The PCR cycling parameters were: 50°C for 2 minutes, 95°C for 10 minutes, followed by 40 cycles of 95°C for 15 seconds and 60°C for 1 minute. All experiments (including negative controls) were performed in triplicate and repeated at least twice. Gene expression levels normalized to endogenous control gene expression were analyzed using the ΔΔCt method and expressed as mean percentages ± SEM relative to the WT condition.

### Immunoprecipitation

Brain tissue or transduced HEK293T cells were extracted in RIPA containing 0.l% SDS and protease inhibitor cocktail (Roche). Lysates were pre-cleared by incubation with PureProteome protein-G magnetic beads (Millipore) rotating at 4°C for 2 h. Mouse monoclonal anti-V5 (Invitrogen) (5 g) was added to the reaction, and the mixture was incubated overnight at 4°C. Beads were then separated using a magnetic stand, and the separated beads were washed 5 times with RIPA 0.1% SDS containing proteinase inhibitors. Bound protein was eluted by incubation of the beads at 95°C for 5 minutes in SDS-PAGE loading buffer with DTT. Using a standard volume for each gel well, samples were loaded from input, eluate, flowthrough, and wash fractions were loaded onto a 10% Bis/Trisacrylamide gel (BioRad), separated, transferred to nitrocellulose membrane, and incubated with anti-Thap1 or anti-V5 antibodies, as indicated. Loaded protein was visualized with Ponceau S stain.

### DNA affinity chromatography

Nuclear extracts of tissue samples and cells were used as inputs for DNA-protein binding purification. Forward and reverse oligos with repeated THABS binding sequence [3,11] were prepared from the forward sequence and biotinylated at the 5′ end:

Fwd:5′-/5Biosg/AGCAAGTAAGGGCAACTACTTCATAGCAAGTAAGGGCAACTACTTCAT-3′. Rv:5′-ATGAAGTAGTTGCCCTTACTTGCTATGAAGTAGTTGCCCTTACTTGCT-3′. Oligonucleotides (20 μM) were annealed by heating to 95°C in buffer (2 mM Tris, pH 7.5, 1 mM MgCl2, 5 mM NaCl, 0.1 mM DTT in DNase free water), and then allowed to cool gradually overnight. Protein samples (1 mg) were pre-cleared for 15 minutes at 4°C using streptavidin agarose resin beads (Thermo) in binding buffer (50 mM HEPES pH 7.9, 150 mM KCl, 6% glycerol, 2.5 mM DTT, 1 mM PMSF, 0.5 mM EDTA, protease inhibitor cocktail (Roche). For protein-DNA binding, samples were incubated with 200 μg of poly(deoxyinosinic-deoxycytidylic) acid (Sigma) for 15 minutes at 4°C, then incubated with the biotinylated oligonucleotides for 2 hours at 4°C. Streptavidin agarose resin beads (Thermo) were added and incubated for 2 hours at 4°C. The resin was washed in binding buffer 5 times, and bound proteins were eluted by heating in DTT-supplemented SDS-PAGE sample buffer for 5 minutes. The supernatant proteins were separated using SDS-PAGE.

### Mass spectrometry sequencing

The proteins eluted from the THABS DNA affinity chromatography column were subjected to SDS-PAGE, and the gel was fixed in a solution containing acetic acid and methanol prior to staining in Colloidal Coomassie Brilliant Blue solution. The desired protein species was identified by alignment of the gel with an aliquot of the eluate blotted with anti-Thap1 and/or anti-V5 antibodies. The 32 kDa or 50 kDa Thap1 species was isolated, and the corresponding gel piece was excised and analyzed by mass spectrometry after trypsin digestion. The analysis was performed using reversed-phase LC over a Waters BEH130 C18 column (75 μm × 150 mm, 1.7 μm particle size) in a Waters NanoAcquity UPLC system (Waters, Milford, MA) interfaced to a Thermo LTQ-Orbitrap mass spectrometer (Thermo Scientific, San Jose, CA).

### Generation of Thap1 KO mice

A detailed description of the generation of Thap1 knockout, i.e. null, mouse is provided in Ruiz *et al.* (unpublished data). Wild type and null alleles were identified by PCR analysis of genomic tail DNA (REDExtract-N-Amp Tissue PCR kit; Sigma, St Louis, MO) with the following primers: Forward GCGTATAATTCAGGCTGTCAG and Reverse GCATTCACCCAAAGCCAATGC. Product sizes were: wild type, 779 bp; KO 254 bp). Embryos at ages E9-12 were collected and used to extract nuclear and cytoplasmic fractions from head and body.

### Statistical analysis

Statistical analysis of RT-qPCR expression levels and densitometry levels for Western blot experiments were analyzed using unpaired Student t-tests. GraphPad software (GraphPad prism 5; San Diego, CA, USA) was used for the analysis, and statistical significance was deemed to be achieved if *p* < 0.05.

## Results

### Characterization of commercial anti-Thap1 antibodies and authentication of endogenous and Thap1-cDNA- overexpression-dependent cellular species in HEK293T cells and primary striatal neurons

**Figure 1:**
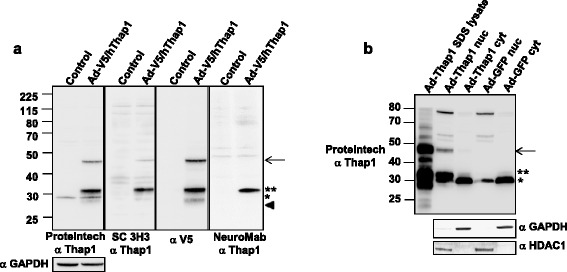
**Transduction of HEK293T cells with Thap1 results in appearance of T1-LIR species of 27 kDa, 32 kDa, and 47 kDa. (a)** HEK293T cells were transduced with adenovirus (Ad)-V5-Tagged human (h)Thap1 at MOI = 5. An aliquot (50 μg) of protein was analysed following separation in a 10% SDS-polyacrylamide gel, transfer, and immunoblotting with each of the following primary antibodies: Proteintech anti-Thap1 (far left panel); Santa Cruz anti-Thap1 antibody SC 3H3 (second panel from left); anti-V5 (third panel from left); NeuroMab anti-Thap1 (right panel). All 4 antibodies recognized a prominent species at 32 kDa (denoted as **) that was not present prior to transduction. Three of the antibodies (excluding the NeuroMab antibody) recognized a transduction-dependent species at 47 kDa (denoted by the arrow). A 29 kDa species (denoted by *) was detected by the Proteintech antibody before and after transduction. The absence of an increase in the intensity of the 29 kDa species recognized by the Proteintech antibody implies that this species is either wholly or partially attributable to a non-Thap1 cross-reacting species. Anti-V5 antibody recognized a species at 27 kDa (denoted by the arrowhead). **(b)** An aliquot (50 μg) of whole SDS protein extract or either nuclear (nuc) or cytoplasmic (cyt) fractions from HEK293T cells transduced with Ad-hThap1 or Ad-GFP control were separated and immunoblotted with either: (1) Proteintech anti-Thap1; (2) anti-GAPDH (cytoplasmic marker); or (3) anti-HDAC1 (nuclear compartment marker). Following fractionation, the 32 and 47 kDa T1-LIR species [symbols as in (a)] are primarily distributed to the nuclear fraction.

**Figure 2:**
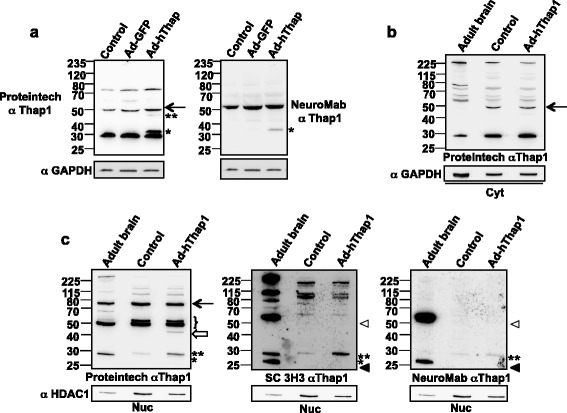
**Primary striatal neurons contain endogenous 32 and 50 kDa T1-LIR species in the nuclear compartment, and the 32 kDa species increases following transduction with Ad-hThap1. (a)** Total cellular extract (50 μg) from untreated (control) mouse striatal neurons blotted with the Proteintech antibody reveals endogenous 29, 50 and 75 kDa T1-LIR species. Following transduction with Ad-hThap1, a transduction-dependent 47 kDa species is detected (**), the 50 kDa species increases slightly in intensity (arrow) and a prominent 32 kDa species (*) appears, as detected by the Proteintech and NeuroMab antibodies. **(b)** An aliquot (40 μg) of protein from the cytoplasmic compartment from adult mouse brain and untreated and Ad-hThap1-transduced primary striatal neurons was separated and immunoblotted as indicated. The 29 kDa T1-LIR species was prominent in all 3 samples, whereas the 50 kDa (arrow) was detected only in primary striatal neuronal extract. T1-LIR species of greater apparent M_r_ were present in all 3 samples. **(c)** The 29 (*), 50 doublet (brackets) and 75 kDa (arrow) T1-LIR species were detected in 10 μg of adult brain nuclear extract, whereas, in primary striatal neuronal extract, a 32 kDa T1-LIR (**) was detected and the upper band of the 50 kDa T1-LIR species was relatively more prominent (brackets). Note that the NeuroMab and Santa Cruz antibodies do not detect all the identical species, but all recognize the 32 kDa species (**), which was observed to increase following transduction with Ad-hThap. The transduction-dependent 47 kDa species is recognized only by Proteintech (empty arrow). SC3H3 and NeuroMab antibodies also recognize a 27 kDa T1-LIR species in adult brain (filled arrowhead) and a very prominent 50+ kDa species (empty arrowhead).

**Figure 3:**
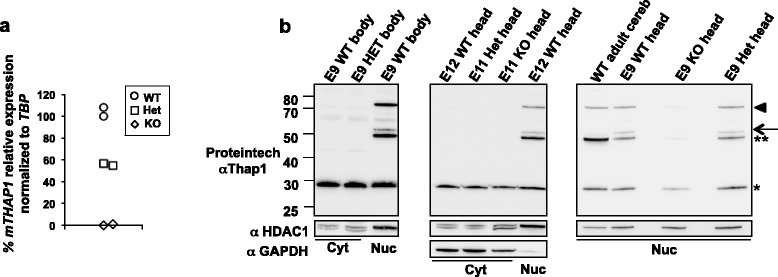
**Levels of Thap1 T1-LIR 32, 50, and 75 kDa species are dramatically reduced in the nuclear compartment of** ***Thap1*** **-null embryos. (a)** RT-qPCR was performed on mRNA derived from whole embyos, E9 Thap1 WT, heterozygote null and homozygote null. *mTHAP1* levels were normalized to *TBP*, and WT was arbitrarily set at 100%. **(b)** Immunoblots of cytoplasmic (cyt, 20 μg) and nuclear (nuc, 10 μg) extracts from E9-E12 *Thap1* ^*+/+*^(WT), *Thap1* ^*+/−*^ (HET) and *Thap1* ^*−/−*^ (KO) embryos following separation into “head” and “body” were performed using the Proteintech antibody. Only the 29 kDa T1-LIR species was observed in the cytoplasmic compartment from E9 *Thap1* ^*+/+*^ and *Thap1* ^*+/−*^ body, whereas the 29 kDa, 50 kDa, and 75 kDa species were observed in the nuclear compartment from these samples (left panel). The level of the 29 kDa species in the cytoplasm was not reduced in the null mutant (middle panel), but the levels of the 29 kDa species (*), the 50 kDa doublet (** and arrow), and 75 kDa (arrowhead) species were markedly reduced in the nuclear compartment of the null embryo “head”, with a greater relative reduction in the higher M_r_ species than in the 29 kDa species (*) (right panel) (representative of 3 null embryos).

**Figure 4:**
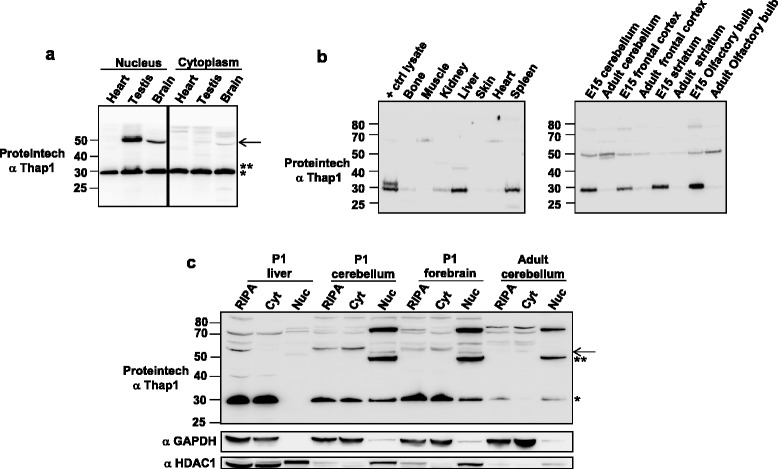
**A 50 kDa T1-LIR species is detected by Proteintech antibody in mouse neural tissue and testes and is developmentally regulated. (a)** Nuclear (150 μg) and cytoplasmic (150 μg) extracts from adult heart, testis, and whole brain were subjected to SDS-PAGE and immunoblotted with Proteintech anti-Thap1 antibody. The 29 kDa T1-LIR species (*) was detected in both compartments in all three tissues. The 32 kDa species (**) was detected only in nuclear extract from testis. The 50 kDa species (arrow) was detected in the nuclear compartment in testis (doublet) and brain, with a trace amount detected in cytoplasm, likely due to contamination with nuclear proteins (not shown). **(b)** Total cellular extracts (50 μg) from adult peripheral organs (left panel) and adult and E15 individual brain regions (right panel) were immunoblotted with Proteintech anti-Thap1 antibody. “Positive control lysate” lane corresponds to HEK293T cells transfected with a human Thap1 expression vector. **(c)** Extracts from liver, cerebellum and forebrain from P1 mice were subjected to SDS-PAGE, immunoblotted, and patterns were compared to those observed in adult cerebellum. Total cellular RIPA (150–200 μg), cytoplasmic (cyt) (150–200 μg) and nuclear (nuc) extracts (50–75 μg) were immunoblotted with Proteintech antibody. The lower 50 kDa T1-LIR species (**) was detected only in brain tissues and the levels of the upper 50 kDa (arrow) and lower 50 kDa were increased in P1 relative to adult. Note that the 50+ kDa species in P1 liver nucleus does not co-migrate with either of the two brain species. The 29 kDa species (*) appeared in nuclear extract only in neural tissue.

Commercial anti-Thap1 antibodies from Proteintech, Inc. (rabbit polyclonal), NeuroMab (mouse monoclonal), and Santa Cruz Biotechnology Inc. (SC3H3, mouse monoclonal) were employed for this study. The identical Proteintech antibody was utilized by LeDoux and colleagues [19], enabling us to compare our data with those collected in that independent study. Our first goal was to determine which, if any, of the proteins identified by the Proteintech, NeuroMab, and/or Santa Cruz antibodies were likely to have identity with authentic Thap1. We focused on Thap1-like immunoreactive species (T1-LIR) of 27, 29, 32, 47, 50 and 52 kDa in HEK293T cells, primary striatal neurons, and mouse peripheral and neural tissues. We determined for each of these T1-LIR whether it was detected endogenously, after transfection/transduction, or both. Following introduction of the T1-LIR species and the systems in which they are visualized in Figures 1, 2, 3 and 4, the T1-LIR species, their authenticity as endogenous Thap1, and the antibodies which recognize them are summarized in Table 1.

**Table 1:** **Distribution of T1-LIR species in HEK293T cells, primary striatal neuronal cultures and murine peripheral and nervous tissues**

**T1-LIR**	**Proteintech**	**NeuroMab**	**Santa Cruz**
27 kDa		Adult brain (N) (Figure 2)	Adult brain (N) (Figure 2)
29 kDa (specific)	Neural structures (N) (Figures 2, 3, 4 and 6)		
29 kDa (non-specific)	HEK293T (R, C, N) (Figures 1, 5 and 7), all mouse organs (R, C) (Figure 4)		
32 kDa	Transduced HEK293T (R, N, C) (Figures 1, 5 and 7) Primary striatal neurons (N) (Figures 2 and 6), testis (N), heart (N) (Figure 4)	Transduced HEK293T(R) (Figures 1 and 5) Primary striatal neurons (N) (Figure 2)	Transduced HEK293T(R) (Figure 1) Primary striatal neurons (N) (Figure 2)
47 kDa	Transduced HEK293T (R, N) (Figures 1 and 7) Transduced primary striatum (R, N) (Figure 2)		Transduced HEK293T (R) (Figure 1)
50 kDa	All neuronal structures, adult, embryonic and P1, and thesis (R, N) (Figures 2, 3 and 4)		
50 kDa (non-specific)		Adult brain (N) (Figure 2)	Adult brain (N) (Figure 2)
52 kDa	All neuronal structures, adult, embryonic and P1, and testis (R, N) (Figures 2, 3 and 4)		Adult cerebellum (N), E9/E12 head (N) (Figure 2)

Whole extract RIPA (R), nuclear extract (N), cytoplasmic extract (C). NI = Non-infected.

In untreated HEK293T cells, the Proteintech antibody detected an endogenous T1-LIR at ~29 kDa (Figure 1a, Proteintech panel, left lane). We transduced HEK293T cells with an adenovirus expressing human Thap1 (Ad-V5hThap1) fused to the epitope tag V5 (Figure 1a, all panels, right lanes). Following transduction with Ad-V5/hThap1-GFP virus, Proteintech anti-Thap1 antibody again recognized an endogenous ~29 kDa species, which increased only slightly in intensity relative to GAPDH (Figure 1a, Proteintech panel, right lane), leading us to conclude that this T1-LIR is largely comprised of a cross-reacting protein and not authentic Thap1. All three anti-Thap1 antibodies recognized a transduction-related, major species of ~32 kDa that was also detected by anti-V5 antibody (Figure 1a, all panels, right lanes). The Proteintech, SC 3H3, and anti-V5 antibodies also recognized a discrete, transduction-related species at ~47 kDa, but the NeuroMab antibody failed to recognize this species (Figure 1a, right lane, all panels). Taken together, these results indicate that three commercial anti-Thap1 antibodies recognize authentic transduced Thap1 at ~32 kDa. Two of the three anti-Thap1 antibodies, and the anti-V5 tag, also recognized a ~47 kDa species that we tentatively identified as posttranslationally modified Thap1. Only the Proteintech antibody recognized an endogenous T1-LIR at ~29 kDa.

In addition to the ~32 kDa T1-LIR species, anti-V5 antibody detected a species of ~27 kDa that was absent from non-transduced lanes (Figure 1a, Proteintech and anti-V5 panel). The specificity of the anti-V5 antibody suggests that, in all likelihood, this ~27 kDa species represents authentic immature, i.e. unmodified, Thap1.

We employed subcellular fractionation to assess the compartmental localization of the T1-LIR species in HEK293T cells. We separated the nuclear and cytoplasmic fractions from lysates of HEK293T cells transduced with Ad-V5/hThap1-GFP or Ad-GFP control (Figure 1b). Both the ~32 kDa as well as the ~47 kDa species were preponderantly located in the nuclear fraction (Figure 1b). Interestingly, the endogenous ~29 kDa T1-LIR species was detected preferentially in the cytoplasmic fraction, but was also detectable in the nuclear fraction (Figure 1b). Notably, an endogenous ~32 kDa species appeared in the cytoplasmic fraction of the Ad-GFP-transduced cells (Figure 1b, far right lane).

We applied this same overexpression strategy to characterization of T1-LIR species in primary murine striatal neuronal cultures, using adenoviral particles into which were packaged untagged human Thap1 cDNA (Ad-hThap1) which also expressed GFP. Notably, the Proteintech antibody recognized a ~29 kDa, a ~50 kDa, and a ~75 kDa species in total cellular extracts of untreated neurons (Figure 2a). We conclude that the ~29 kDa T1-LIR is what we had previously seen at ~30 kDa [15]. Transduction with Ad-hThap1 was associated with a relatively greater increase in the level of the ~32 kDa species than in that of the other species. Low levels of a ~47 kDa species were observed exclusively in transduced cells (Figure 2a). The M_r_ of that species raised the possibility that this protein may be related to the ~47 kDa species that we observed following overexpression of Thap1 in HEK293T cells (Figure 1a). The 47 kDa T1-LIR bands co-migrate in a side-by-side comparison of primary striatal neurons and HEK cells over-expressing Thap1 (data not shown). Additionally, we observed a small increase of the endogenous 50 kDa T1-LIR species upon transduction with Ad-Thap1, compared to Ad-GFP control vector (Figure 2a). Once again, the NeuroMab antibody recognized the ~32 kDa transduction-dependent protein, but otherwise did not recognize the same T1-LIR as the Proteintech antibody.

We repeated the transduction of primary neurons with nuclear/cytoplasmic partitioning. We also used this experiment to compare T1-LIR species detected by the three antibodies in primary neuronal cultures and adult brain (Figure 2b). None of the 3 antibodies detected an obvious increase in T1-LIR in the cytoplasmic fraction following transduction (Figure 2b) (NeuroMab and SC3H3, not shown). All three antibodies detected an endogenous, nuclear ~32 kDa species, i.e. a species slightly larger than the ~29 kDa species that Proteintech recognizes in cytoplasmic fractions or total cellular extracts or in HEK293T cells. The level of this species increased following transduction. A species of similar M_r_ was detected in adult brain nuclear extract, particularly with SC3H3. The three antibodies all recognized species at 50 kDa and above in adult brain, but, when the patterns of T1-LIR were compared, there was no consensus detection of any major species at this higher molecular weight (Figure 2c). The Proteintech antibody recognized a species in the nuclear fraction at ~50 kDa which appeared as a doublet (i.e. two T1-LIRs very close in size to 50 kDa), likely due to different posttranslational modification, and very similar in adult brain and primary striatal neurons (Figure 2c, left panel). This antibody also again detected a transduction-dependent ~47 kDa that localized to the nuclear fraction. In adult brain, the SantaCruz and NeuroMab antibodies recognized a very prominent nuclear species of M_r_ ~55-60 kDa. The SantaCruz antibody recognized multiple species of even higher M_r_, as did the Proteintech antibody. Both SantaCruz and NeuroMab antibodies also recognized an endogenous ~27 kDa species in adult brain nuclear extract (Figure 2c), mid and right panel). These species of M_r_ > ~50 kDa in primary striatal neurons, were not increased by overexpression of Thap1. From these data, we concluded that the endogenous ~32 kDa species likely represents authentic Thap1, but these data were insufficient to draw any conclusions regarding the species with M_r_ equal to or greater than 50 kDa, which required and required additional assays.

### Mass spectrometric confirmation of the identity of the ~32 kDa species as authentic Thap1

Tryptic digests of the ~32 kDa T1-LIR species were subjected to mass spectrometry analysis. This procedure yielded two peptides:

- Peptide 1: IHQLEQQVEK
- Peptide 2: EVVHFQKEKDDVSERGYVILPNDYFEIVEVPA-COOH

Peptide 1 is an internal Thap1 sequence, while Peptide 2 represents the carboxyl terminal 32 amino acids of Thap1. This represents the first definitive identification of physiologically generated Thap1. Methylation of the evolutionarily conserved glutamate at position E157 (underlined) was detected in this assay system, and importantly, this residue is within the nuclear localization signal (NLS) [24]. Although the posttranslational modification of glutamate by methylation was first recognized in prokaryotes, this posttranslational modification in eukaryotes has been identified to play roles in protein folding and trafficking [25]. Given its location within the NLS of Thap1, one might speculate that the methylation status controls utilization of the NLS to import Thap1 into the nucleus. Evaluation of this hypothesis is in progress.

### Analysis of Thap1-LIR species in Thap1 null embryos

In order to determine which endogenous T1-LIR species were most likely to have identity with authentic Thap1, we generated a mouse model with a *Thap1*-null allele (Ruiz, Ozelius and Ehrlich, unpublished data). The homozygous null genotype was early embryonic lethal prior to E8, but rare embryos survived up to age E12.5. RT-qPCR of mRNA from null embryos displayed elimination of m*Thap1* mRNA (Figure 3a). Using the Proteintech antibody, cytoplasmic fractions from both head and body showed only the endogenous T1-LIR species of ~29 kDa, whereas nuclear compartments showed the ~29 kDa species and the aforementioned doublet at 50–52 kDa and a species at higher Mr of 75 kDa (Figure 3b), left panel). *Western blot analysis of endogenous mouse Thap1 (mThap1) species in the cytoplasmic fraction extracted from the heads of E11 embryos revealed no genotype-dependent differences (Figure**3**b), middle panel).* These results led us to conclude that, similar to HEK293T cells, the cytoplasmic species of ~29 kDa was largely, if not exclusively, composed of cross-reacting proteins that were not authentic Thap1.

Nuclear fractions extracted from the heads of E9-E12 mutant embryos and probed with Proteintech anti-Thap1 antibody revealed a drastic reduction of the ~50/52 kDa T1-LIR doublet and the ~75 kDa species, and a very noticeable, but lesser, reduction of the ~29 kDa species (Figure 3b, right panel, n = 3). Comparison of the nuclear and cytoplasmic fractions from either “head” or “body”, each of which contains neural and non-neural tissue, indicated that the ~50 kDa T1-LIR species was observed exclusively in the nuclear fraction. Interestingly, the upper species of the 50 kDa doublet detected by Proteintech antibody was more prominent in embryonic tissue (head and body) than in adult cerebellum (Figure 3b). Finally, a T1-LIR species of M_r_ at just over 50 kDa as detected by SC3H3 was also dramatically reduced in the nuclear extract from homozygous null embryo head (Figure 3c). This species is present in the WT adult cerebellum and overlaps with the upper 50 kDa species revealed by Proteintech anti-Thap1. The amount of protein obtained from the E9 embryos was not sufficient to identify T1-LIR species in these samples by NeuroMab antibody (not shown), due to low detection level of specific endogenous Thap1 bands by this monoclonal antibody. There was no T1-LIR detected by NeuroMab or SC3H3 which was definitively decreased or absent in the null embryo. Again due to limited material from the embryos, the amount of nuclear extract is probably insufficient to detect the ~32 kDa species previously found in primary cultures (Figure 2b), middle panel). The proof of the efficacy of a “null”, or “knockout”, allele in the mouse is generally the complete elimination of the protein produced from transcription and translation of that gene. In this study, there was no single species detected by the three antibodies that was completely abolished from the immunoblots of homozygote null embryos. As noted above, Thap1 is a member of a large family of protein with homologous regions. The homozygous lethality combined with the elimination of Thap1 mRNA and the dramatic but incomplete reductions in the ~50 kDa T1-LIR doublet and the nuclear ~29 kDa species led us to conclude that authentic Thap1 was co-migrating with cross-reacting species but that a major component of these species as recognized by the Proteintech antibody represent authentic Thap1. Given the preponderance of the data, this explanation would be the most parsimonious.

### Survey of T1-LIR species in peripheral tissues and brain regions

A published survey of adult rat brain regions by RT-qPCR and *in situ* hybridization [19] showed that the highest level of Thap1 mRNA was detected in the cerebellum with a lower and relatively equivalent level detected across all other brain regions. Developmentally, with the exception of cerebellum, Thap1 mRNA level reached a maximum in all rat brain regions at P7 after which the mRNA levels declined to those observed in adult brain. In the BioGPS databank of gene expression (www.biogps.org), the highest expressing tissues in human are heart, muscle, and liver. Amongst brain regions, caudate and cerebellum express the lowest levels of Thap1 mRNA. In mouse, levels of Thap1 mRNA in testes were at least 5-fold higher than those observed in other tissues.

We performed tissue and region surveys of T1-LIR based on the reported mRNA distribution. Extracts from nuclear and cytoplasmic fractions of heart, testis, and brain were analyzed following overloading of SDS-PAGE gels (Figure 4a). With Proteintech anti-Thap1, the ~29 kDa species was present in all three tissues and in both compartments. A small amount of the ~32 kDa species was observed in the nuclear compartment from testis, and an even lesser amount in heart. T1-LIR species of M_r_ ~ 50 kDa were identified in nuclear fractions from testis and brain, but not heart.

We then performed a survey with total cellular extracts from peripheral organs (Figure 4b, left panel). T1-LIR species of ~50 kDa was not detected. The “positive control lysate” lane, which corresponds to HEK293T cells transfected with 250 ng of hThap1 cDNA, (Figure 4b, left panel, left lane) contains both the endogenous ~29 kDa protein and the transfection-dependent ~32 kDa protein. Total cellular extract from E15 and adult brain regions were assayed (Figure 4b, right panel). All brain regions contained one or more T1-LIR species at ~50 kDa which varied somewhat in intensity with age and region, and which was more prominent in regional extracts than in total brain. The ~29 kDa species was much higher in level at E15 as compared with adult. Comparing nuclear and cytoplasmic fractions from P1 and adult tissues (Figure 4c), the ~50 kDa T1-LIR species were again always located in nuclear fractions, were absent from P1 liver, and decreased in level from P1 to adult in cerebellum. Conversely, the cytoplasmic fraction of these dissected CNS regions contained the ~29 kDa T1-LIR species (Figure 4c). *From these experiments, we conclude that, at all ages, the ~50 kDa T1-LIR species are exclusively present in neural tissue and testes, where they are located in the nuclear fraction and where their levels are developmentally regulated.*

A summary of all the T1-LIR detected by the three different anti-Thap1 antibodies in HEK293T cells and primary striatum cultures, untreated or transduced, and in all murine peripheral organs and CNS regions at different developmental stages is collected in Table 1. The cell compartment where each Thap1 isoform was detected is also indicated. Note that the 50 kDa band detected by Proteintech antibody in neural structures and testis is not equivalent to the non-specific 50 kDa band detected by NeuroMab, the latter of which is a cross-reactive protein. Parallel to this situation, the 29 kDa band detected by Proteintech in total cellular extract is composed both by non-specific protein, located mostly in the cytoplasmic compartment, but also in the nucleus at lower concentrations, and by authentic 29 kDa Thap1 species which overlaps at the same M_r_ and can be detected in primary striatum cultures and murine CNS regions. Importantly, all three antibodies recognize species that are not authentic Thap1, and the Proteintech antibody appears to be the most sensitive for the identification of authentic Thap1.

### siRNA and shRNA knock-down of T1-LIR transduced species in HEK293T cells

**Figure 5:**
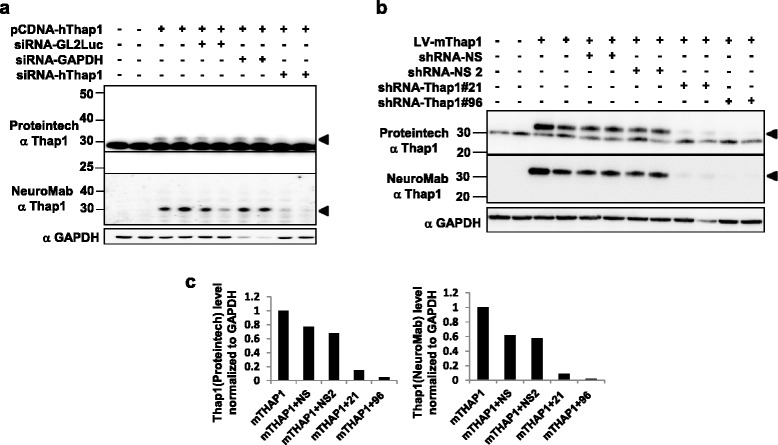
**Multiple Thap1 siRNA and shRNA sequences decrease level of the transfection- or transduction-dependent mouse and human 32 kDa T1-LIR in HEK293T cells. (a)** HEK293T cells were transfected with pCDNA-hThap1 alone or combined with siRNA sequences specific to hThap1 (siRNA 1-hThap1), an unrelated cDNA sequence (GL2Luc), and/or an shRNA targeting GAPDH. Total cellular extracts (15 μg) were immunoblotted with Proteintech anti-Thap1 (top panel) or NeuroMab anti-Thap1 (bottom panel). **(b)** HEK293T cells were transduced with LV-mThap1 alone or together with shRNA sequences, #21 and #96, directed at mouse Thap1, or an shRNA non-silencing control (NS). Total cellular extracts (15 μg) were immunoblotted with Proteintech (top) or NeuroMab (bottom) anti-Thap1 antibodies. **(c)** Levels of 32 kDa Thap1 silencing in Proteintech and Neuromab immunoblots from **(b)**, normalized to GAPDH. Control = untreated cells. Each graph is representative of 2 independent experiments.

To further establish the authenticity of the T1-LIR species and to identify reagents which will be useful for functional studies, we co-transfected HEK293T cells with an expression vector carrying untagged hThap1 cDNA (pCDNA6.1-hThap1) either alone or in combination with ON-TARGET siRNA specific to hThap1 (Dharmacon), GAPDH siRNA, and/or a nonspecific GL2-Luc control [18,26] (Figure 5(a). Co-transfection of the Thap1 siRNA sequences virtually abolished the ~32 kDa T1-LIR species that appeared following expression of pCDNA6.1-hThap1. The ~29 kDa endogenous T1-LIR species did not noticeably decrease, consistent with our conclusion that as detected in total cellular extract or cytoplasm, it is largely a co-migrating protein that is not authentic Thap1. We were unable to explain the decrease in transfected Thap1 in Lane 6. Unlike the observations in Zhao *et al.* [19], in our hands, we were unable to knock down any of the human T1-LIR species with the hTHAP1-RNAi s30274 from Ambion (data not shown), perhaps due to protocol differences.

As we were most interested in performing our studies in mouse neurons, we established the efficacy of reagents in HEK293T cells expressing transduced mThap1. Co-transfection of a lentiviral vector carrying mThap1 cDNA (LV-mThap1-GFP) together with the lentiviral vectors carrying the mThap-1 specific shRNA#21 or shRNA#96 sequences knocked down the ~32 kDa T1-LIR species detected by Proteintech and NeuroMab antibodies in HEK293T. Signal was reduced by either 80% (shRNA#21) or 95% (shRNA#96) compared to non-silencing shRNA control (Figures 5b and 5c).

### Viral-mediated shRNA knock-down of T1-LIR transduced and endogenous species in primary striatal neurons

**Figure 6:**
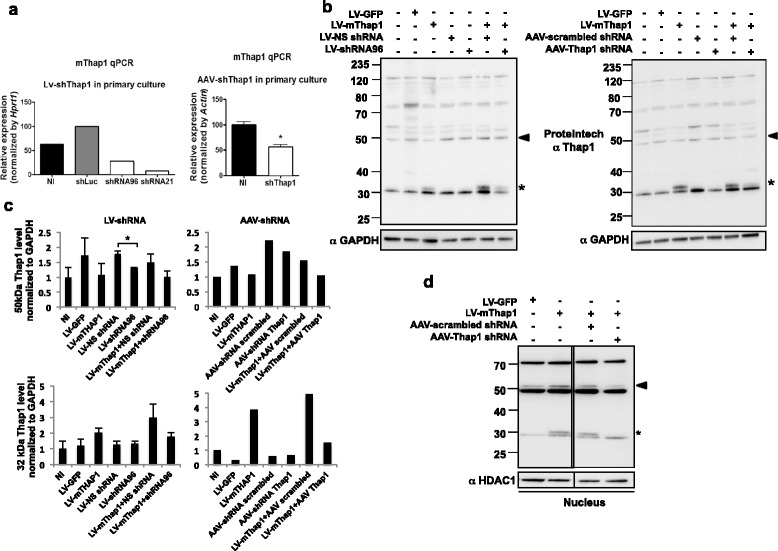
**Silencing of Thap1 in mouse primary striatal neurons leads to a decrease in the levels of the 32 kDa and 50 kDa T1-LIR species. (a)** Quantitative real-time PCR assay of mTHAP1 was performed on cDNA derived from mRNA from untreated mouse primary striatal neurons and following transduction with lentiviral (LV) particles expressing mThap1, shThap21 or 96, or shLuc control sequences (left panel) (representative of 2 independent experiments) or AAV particles expressing a third shRNA sequence directed at mThap1 (N = 3) (Unpaired *t-test*: *p < 0.05; Untreated level arbitrarily set at 100%). **(b)** Left panel: Westem blotting of total cellular extract, 40 μg, from primary striatal neurons transduced with either LV-mThap1 or LV-GFP control alone or together with lentivirus carrying shRNA #96 sequence directed at mThap1 or non-silencing (NS) shRNA. Right panel: Identical experiment as in left panel except for replacement of LV with AAV with a different shRNA sequence, as in **(a)**. MOI for LV = 2.5 viral particles/neuron; for AAV = 100,000 viral particles/neuron. **(c)** Graphs show densitometry of blots from experiments represented in **(b)**. (N = 4 for LV-4, shRNA, N = 2 for AAV-shRNA). (NI = non-infected) **(d)** Transduction of primary striatal neurons with LV-mThap1 increases the intensity of the 32 (*) and upper 50 kDa (arrowhead) T1-LIR species in nuclear extracts (10 μg), and the relative increase is decreased by co-transduction with AAV-shRNA specific to Thap1 in the nucleus of primary striatum cultures.

To transduce primary striatal neurons in culture, we generated lentiviral particles carrying either mouse Thap1 (LV-mThap1-GFP) or pGIPZ-shRNA (#21 and #96, described above) specific to mouse Thap1, and AAV particles carrying a third shRNA sequence (Virovek). We observed that transduction with LV-shRNA (#96), LV-shRNA (#21), or AAV-shRNA alone led to a significant decrease in mThap1 mRNA as assayed by RT-qPCR (Figure 6a, left and right panels). For all three silencing vectors, particularly #21, multiplicity-of-infection (MOI) and time to harvesting after transduction were limited by cell death, leading us to hypothesize that complete elimination of Thap1 may be lethal to primary, post-mitotic neurons.

At the protein level, transduction with LV-shRNA (#96) (Figure 6b, left panel) or AAV-shRNA (Figure 6b, right panel) together with LV-mThap1 efficiently knocked down or completely eliminated the ~32 kDa transduction-related species. In total cellular extracts, we observed an obvious knockdown of the transduced ~32 kDa species (40% reduction using LV-shRNA and 69% reduction using AAV-shRNA; Figures 6b and 6c) as well as knockdown of the ~50 kDa complex by 30% (Figures 6b and 6c). We attempted to knock down the 50 kDa species to a greater extent by increasing the MOI and/or longer incubations with the shRNA lentiviral particles; however, as mentioned above, MOI over 10 for LV treatment or harvesting time points later than 72 h was associated with severely compromised survival of the cultured neurons.

As the separation of subcellular compartments led to improved detection of the transduction-dependent Thap1-LIR 32 kDa species and to resolution of the 50 kDa doublet in the nuclear compartment (Figure 2b, left panel), we repeated co-transduction of LV-mThap1-GFP with either AAV-shRNA or LV-shRNA#96 followed by fractionation of cells into cytoplasmic and nuclear compartments. In the nuclear fraction, we observed a complete knockdown of the ~32 kDa species with AAV-shRNA and a 50% decrease in the ~50 kDa species (all reductions are expressed relative to the corresponding levels of the proteins observed in the presence of control non-silencing shRNA; Figure 6d). The maximum tolerated MOI (100,000 for AAV) was again a limiting factor.

**Figure 7:**
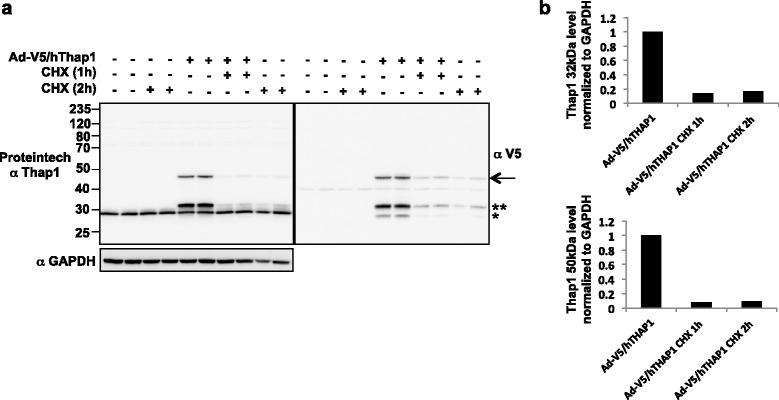
**The 32 kDa and 47 kDa T1-LIR species have half-lives of less than two hours. (a)** The half-life of the 32 kDa (**) and 47 kDa (arrow) transfection-dependent T1-LIR species was calculated by transfection of HEK293T cells with pCDNA-V5/hThap1 and treatment with 50 μg/ml cycloheximide (CHX) 24 h post-transfection. Cells were harvested at the indicated time points and total cellular extracts (35 μg) were immunoblotted with the Proteintech antibody (left panel), followed by the Neuromab antibody (right panel). Each panel is representative of two experiments. Note that the Neuromab antibody again recognizes a 27 kDa species (*), with similar half-life. **(b)** Densitometry of the transfection-dependent 32 kDa and 47 kDa species in the left panel, normalized to GAPDH, and level of transfection-dependent species prior to cycloheximide arbitrarily set to 1.0.

To assess the half-lives of the ~47 kDa and ~32 kDa T1-LIR species, we employed a cycloheximide protocol in HEK293T cells to arrest protein synthesis, enabling us to selectively monitor protein degradation (Figure 7). In total cellular extracts, the transduction-dependent T1-LIR species at ~47 kDa and ~32 kDa had identical half-lives of less than two hours, regardless of whether the degradation was monitored using the Proteintech anti-Thap1 antibody (Figure 7, left panel) or the anti-V5 antibody (Figure 7, right panel). The ~47 kDa T1-LIR species was reduced by 90% and the ~32 kDa species was reduced by 85% after 1 h of protein synthesis inhibition. The ~27 kDa species detected by anti-V5 (Figure 1a, right panel) also decreased after 1 h of cycloheximide treatment. Conversely, the endogenous HEK293T ~29 kDa species detected by Proteintech in total cellular extract was not appreciably degraded during this period, adding further support to the notion that this endogenous ~29 kDa protein is primarily composed of a cross-reactive protein not identical to Thap1. Combined with the shRNA knockdown experiments, these data imply that the endogenous neuronal 50 kDa T1-LIR may be more stable than the transfected 47 kDa species, perhaps due to different posttranslational modifications or to cell-type or species differences between human HEK293T cells and mouse neurons.

### Enrichment of T1-LIR species by immunoprecipitation and DNA affinity chromatography

**Figure 8:**
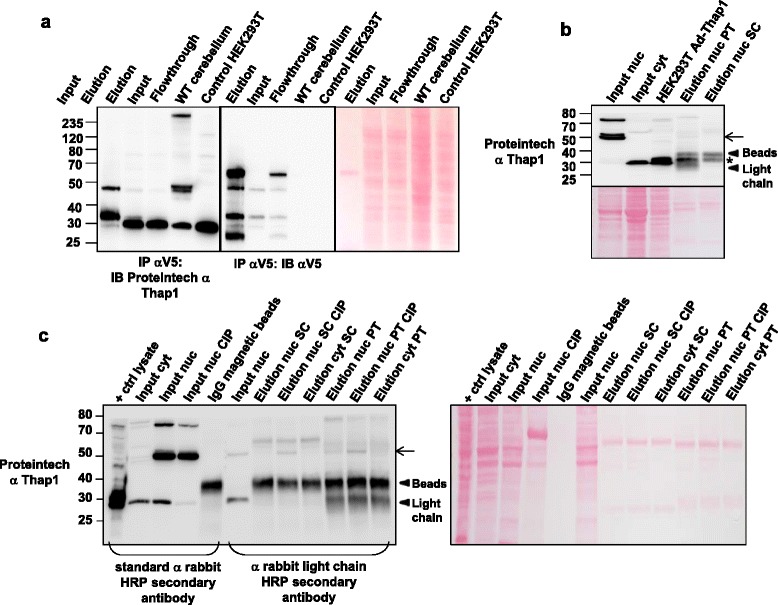
**The T1-LIR 32 kDa and 50 kDa species are enriched by immunoprecipitation (IP) in transduced HEK293T and wild type newborn brain nuclear extract. (a)** Total cellular extracts from HEK293T cells transduced with Ad-V5/Thap1-GFP were subjected to immunoprecipitation using anti-V5 antibody, separated by PAGE, and transferred to nitrocellulose. The membrane was incubated with Proteintech anti-Thap1 and anti-V5 antibody. On the right panel, Ponceau S staining of the membrane shows relative protein loading in each lane. Control = untreated cells. **(b)** Nuclear and cytoplasmic extracts from P1 forebrain were subjected to immunoprecipitation using Proteintech antibody or the SC3H3 antibody. The T1-LIR species of 32 kDa (*) and a very small amount of the 50 kDa species (arrow) can be detected in the elution with the Proteintech (PT) antibody. IP with Santa Cruz 3H3 (SC) (last lane) confirms the enrichment of the 32 kDa species. **(c)** A protocol similar to that employed in **(b)** was used except that the P1 forebrain extract was treated with calf intestinal phosphatase (CIP) prior to IP. IP was perfomed using Proteintech (PT) or Santa Cruz (SC) antibodies. Note that the 32 kDa T1-LIR species is obscured by the light chain in the eluate, but the 50 kDa T1-LIR species (arrow) is more easily identified in nuclear samples treated with CIP (compare to 8b).

Establishment of the ability to immunoprecipitate (IP) T1-LIR species would be important for any study of Thap1 posttranslational modifications. HEK293T cells were transduced with Ad-V5/hThap1-GFP viral particles, and the lysates were subjected to IP with anti-V5 antibody. The anti-V5 immunoprecipitate was then separated in polyacrylamide gels and transferred to nitrocellulose prior to incubation with primary antibodies (Figure 8a). Immunoblotting with the Proteintech anti-Thap1 or anti-V5 antibodies revealed substantial enrichment of the ~32 kDa and ~47 kDa T1-LIR species when compared to the levels of these proteins in the input (Figure 8a). Conversely, the level of the ~29 kDa T1-LIR species was lower in the elution relative to the input after anti-V5 IP, again consistent with our conclusion that this species is largely composed of a cross-reacting species, and not authentic Thap1. Notably, the anti-V5 antibody also shows enrichment of the 27 kDa T1-LIR in the elution, also consistent with our suggestion that this band represents unmodified Thap1.

We also sought to assess the enrichment by IP of the endogenous T1-LIR species from newborn brain nuclear fractions (Figure 8b). The endogenous TL1-LIR ~32 kDa species was highly enriched by IP of mouse newborn brain nuclear extract using either the Proteintech anti-Thap1 or the Santa Cruz anti-Thap1 followed by immunoblotting with Proteintech anti-Thap1 (Figure 8b), but the ~50 kDa T1-LIR was barely detectable. We then treated the brain nuclear extract with calf intestinal phosphatase (CIP) to determine if the doublet at 50 kDa may be related to differences in phosphorylation status (Figure 8c). In fact, with CIP-treated nuclear extract, the upper band of the doublet appeared to decrease in intensity and the 50 kDa species was more apparent after IP, possibly by exposing protein epitopes recognized by Proteintech and SC3H3 antibodies in the 50 kDa species. As the light chain co-migrates with the endogenous 32 kDa species, we were unable to determine how treatment with CIP may or may not have modified IP of this T1-LIR.

**Figure 9:**
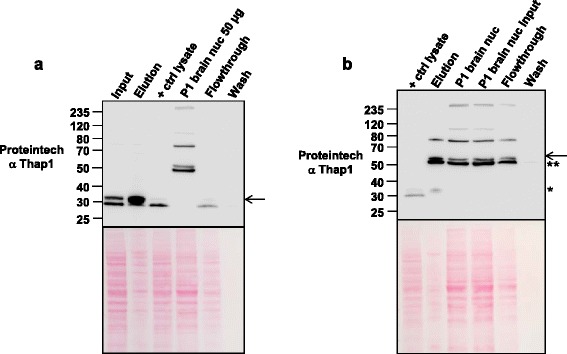
**The T1-LIR 32 kDa (transduced HEK293T and P1 brain) and 50 kDa (P1 brain) species are enriched by oligonucleotide DNA affinity chromatography. (a)** Total cellular extracts from HEK293T cells transduced with Ad-mThap1 were subjected to DNA affinity chromatography using a THABS oligonucleotide polymer. The 32 kDa T1-LIR species (arrow) was enriched in the eluate relative to input. **(b)** P1 forebrain nuclear (nuc) extract was subjected to DNA affinity chromatography using a THABS DNA oligonucleotide column. The endogenous 32 kDa (*) and 50 kDa doublet (** and arrow) T1-LIR species were enriched in the eluate relative to input. Bottom panels are the membranes stained with Ponceau S.

Given the fact that Thap1 is a transcription factor, we sought to determine which of the endogenous T1-LIR species would bind to a Thap1-Binding Sequence (THABS: 5′-TxxxGGCA-3′) [11]. We prepared a column of immobilized THABS sequences expressed in tandem in an oligonucleotide that was biotinylated on the 5′ end to crosslink to streptavidin-sepharose. We performed affinity chromatography using nuclear extract from HEK293T cells prepared from cells overexpressing V5-Thap1 and with nuclear extract of P1 mouse brain. From HEK293T nuclear extract, the transfection-dependent ~32 kDa was highly enriched in the eluate (Figure 9a). From brain, the endogenous ~32 kDa was also highly enriched in the eluate. The ~50 kDa species was consistently retained during the wash but re-appeared with elution and was also modestly enriched in this eluate, as was the 75 kDa species (Figure 9b). These data demonstrate high affinity of the endogenous ~32 kDa, ~50 kDa, and even the ~75 kDa T1-LIR species for immobilized THABS. Binding kinetics and measurements of column capacity will be required in order to determine the K_d_ of the interaction, which will be important in establishing the likelihood that the interaction between these T1-LIR species and THABS occurs *in vivo*.

### Downstream targets

**Figure 10:**
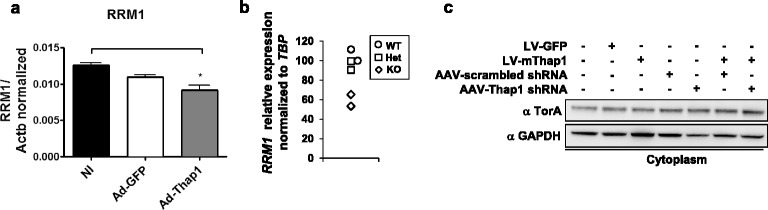
***Rrm1*** **mRNA level is altered by up- or down-regulation of Thap1 but mRNA and protein levels of torsinA are unchanged. (a)** *Rrm1* expression analysis by RT-qPCR in primary striatal neuronal cultures transduced with Ad-mThap1 virus for Thap1 overexpression, or Ad-GFP control. (*p < 0.05; Untreated level arbitrarily set at 100%; N = 3). **(b)** *Rrm1* qPCR analysis in whole body of E9 Thap1-null embryos (diamonds), heterozygous (squares) and wildtype littermates (circles). (N = 2) **(c)** TorsinA protein levels in primary striatal neuronal cultures transduced with LV-mThap1 to overexpress Thap1 protein or treated with AAV-shRNA specific to mThap1.

Lastly, we took advantage of neuronal preparations with overexpression or knock down of Thap1 protein as systems for assaying the effects of increased or decreased Thap1 on the levels of putative downstream targets whose transcription may be inferred to be regulated by Thap1, i.e. *RRM1* [18] and *TOR1A* [15,17]. Using qPCR, RRM1 mRNA was decreased by 33% following transduction of primary striatal neurons with Ad-hThap1 (p < 0.01, N =3), whereas RRM1 mRNA levels were unchanged following transduction with Ad-GFP (Figure 10a). We also assayed RRM1 mRNA level in null embryos (N = 2) and again found a decrease of 30% (Figure 10b). These data are consistent with those of Girard and colleagues [18] who demonstrated similar phenotypes in HUVEC cells following up- or down-regulation of Thap1. We and others have suggested that *Tor1a* may be a Thap1 target [15,17], thereby providing a molecular link between the pathogenesis of DYT1 and that of DYT6. In order to test this possibility, we measured torsinA mRNA and protein following overexpression and knockdown of Thap1. However, there was no change in torsinA mRNA (data not shown) or protein associated with either manipulation (N = 3–6, p > 0.05; Figure 10c).

## Discussion

Dystonias are poorly understood, untreatable causes of substantial disability which occur either as isolated disease entities or as features of other movement disorders, e.g., Parkinson’s disease. The progress in discovering dystonia genes and the characterization of their protein products represent the most promising, rational direction for development of evidence-based interventions aimed at treating or preventing these enigmatic illnesses. Since the discovery of *THAP1* as the gene mutated in *DYT6* dystonia [1-4], very little information has emerged about the biology of Thap1 protein speciation and distribution, particularly in the brain. These data are required for study of the function of the protein and for elucidation of the mechanism(s) via which mutations in *THAP1* cause dystonia. Herein, by using three different commercially available anti-Thap1 antibodies, we present evidence that first, Thap1 is a methylated DNA-binding protein that exists as complex families of proteins of M_r_ ~30 kDa and ~50 kDa. Second, we were able to validate a novel endogenous ~32 kDa and a doublet at 50 kDa, all of which correspond to authentic murine Thap1, despite the limitations of these antibodies. Third, we demonstrated that one of the antibodies (Proteintech) recognizes a nonspecific species at 29 kDa in neuronal and non-neuronal tissue and the other two antibodies (SC3H3 and NeuroMab) recognize a nonspecific species at 50 kDa in adult brain tissue. Importantly, however, there is also a specific ~29 kDa T1-LIR that co-migrates with a non-Thap1 protein. Finally, although we were able to induce ectopic expression of the ~32 kDa and higher M_r_ species in human embryonic kidney cells, the endogenous ~50 kDa doublet was observed exclusively in the nuclear compartments of nervous tissue and testis. Consistent with its identity as authentic Thap1, this species displayed a high apparent affinity for THABs.

Our results contrast with those published by Zhao *et al.* [19]. In addition to the fact that there is a definite size difference in the major T1-LIR species detected in the two studies, our data indicate that the major cytoplasmic T1-LIR identified by the Proteintech antibody is comprised almost entirely of a cross-reacting protein and not of authentic Thap1. Interestingly, in the subcellular fractionation included in that previous study, the nuclear fraction was shown to contain as much tubulin as the cytoplasmic fraction. We suspect, therefore, that immunocytochemical staining of endogenous Thap1 with this antibody is not reliable.

The endogenous ~32 kDa T1-LIR species is much more easily detected in nuclear extracts from embryonic neurons and early prenatal brain than from adult neuronal structures. These data suggest a role for Thap1 in neuronal development. However, this observation does not permit any conclusions as to the specific function of Thap1. Any suggested role in the regulation of cell cycle and/or apoptotic events that accompany neuronal and brain development would be speculative at this time. Of note, however, we were unable to increase or decrease Thap1 protein level in neurons beyond a certain point without cell death, implying tight regulation of its level is required for growth and survival [18]. As the ~32 kDa T1-LIR species is highly enriched by oligonucleotide affinity chromatography from HEK293T cells, we assume that this species is the DNA binding species in peripheral tissues. Testing this hypothesis would require additional similar experiments from multiple peripheral organs in order to draw firm conclusions. These extended experiments are beyond the scope of our current study.

We propose that the most important feature of the current report is the identification of an endogenous, nuclear ~50 kDa doublet species found in the nervous system and in testis but no other organs. Of note, a similar situation exists in DYT3, in which a neuronal specific isoform of the causative protein, TAF1, was identified [27,28]. This novel Thap1 species was most abundant in cerebellum where its levels were obviously developmentally regulated. The existence of a “neuron-specific” Thap1 species that binds DNA has significant implications when thinking about how mutations in a ubiquitously expressed gene result in a neurologic disease without obvious pathophysiology in other organs. Thus, it will be critical to determine the nature and function of this higher M_r_ species, and how its formation and behavior is modified by disease mutations. Although Thap1 has been proposed to dimerize and to bind DNA as a dimer [20,21], there is insufficient data to conclude that it is a covalent bound dimer that would persist following our extraction procedures. Unfortunately, its relatively low abundance and poor immunoprecipitation by the commercial antibodies described herein prevented sufficient recovery for sequencing by mass spectrometry. Thus, although the 50 kDa T1-LIR is virtually eliminated in extract from the homozygote knockout embryo its complete characterization will require additional purification methods in order to recover sufficient material for MS sequencing, including identification of the PTMs. As its M_r_ is not identical to that which is achieved by overexpression of Thap1 in HEK293T cells, i.e. 50 kDa vs. 47 kDa, it will be necessary to isolate these species from neuronal tissue to definitively identify their relationship to Thap1. As noted above, the enrichment of the ~50 kDa species in the nuclei of neurons and evidence that it is the major Thap1 species that binds to DNA, when taken together, suggest that this species may be important in the pathogenesis of *DYT6* dystonia. The fine regulation of the levels of the ~50 kDa species at .different embryonic stages and its prevalence in the CNS versus other murine tissues suggests that this species of Thap1 (along with the 32 kDa species) might also play a role in the development of the CNS, particularly in the striatum and/or cerebellum, one or both of which have been implicated in the pathophysiology of dystonia (reviewed in [29]).

While awaiting further characterization of the 50 kDa species, including the possible association of Thap1 with other protein(s), it is important to consider how posttranslational modifications may be contributing to the heterogeneity in M_r_ of T1-LIR species. The 30/50/70 kDa “ladder” is a pattern highly reminiscent of that generated by SUMOylation [30]. *In silico* analysis of Thap1 protein reveals a consensus SUMOylation site surrounding amino acid K64 and non-consensus sites at positions K90 and K190 (SUMOplot™ Analysis Program). Using a cell-based SUMOylation assay, a portion of Thap1 in transfected HEK293T cells is apparently SUMOylated, as a function of co-transfection of SUMO-1, but not Ubc9 (unpublished observations). Further analysis of potential SUMOylation of Thap1 is required, with particular attention to neurons. Implication and identification of a specific E3 ligase will also be important. Other consensus sites for posttranslational modification include substrates for phosphorylation and glycosylation (Glycosylation servers: NetCGlyc http://www.cbs.dtu.dk/services/NetCGlyc; NetOGlyc http://www.cbs.dtu.dk/services/NetOGlyc; NetNGlyc http://www.cbs.dtu.dk/services/NetNGlyc. Phosphorylation servers: NetPhosK http://www.cbs.dtu.dk/services/NetPhosK/). Indeed, in the results of our immunoprecipitation experiments presented above, a Thap1 epitope was apparently revealed after treatment with CIP. Identification of all posttranslational modifications requires the comprehensive purification and characterization of all T1-LIR species.

The absence of changes in torsinA protein expression upon up- or down-regulation of Thap1 in our system was unexpected, given the evidence demonstrating that the *Tor1A* promoter contains Thap1 binding sites and that *Tor1A* promoter activity is repressed by Thap1 [15,17]. These observations regarding invariable torsinA protein levels in neurons after Thap1 modulation are consistent with the reported absence of mRNA fluctuations upon Thap1 siRNA-mediated knock down in human fibroblasts [17]. Conceivably, there are other regions, conditions, and/or mechanisms via which Thap1 and torsinA could interact, but, at least in the systems tested to date by us and others, there is no obvious evidence supporting the existence of positive or negative regulation of endogenous *Tor1a* by Thap1 protein.

In summary, we present evidence that in addition to other species, Thap1 exists as a nuclear, “neuronal-specific” (other than testis) doublet of ~ M_r_ 50 kDa. The presence of a “neuronal-specific” species has profound implications for how mutations may differentially affect Thap1 function in neurons relative to peripheral organs, thereby leading to a neurologic disease.
